# Rethinking fast and slow based on a critique of reaction-time reverse inference

**DOI:** 10.1038/ncomms8455

**Published:** 2015-07-02

**Authors:** Ian Krajbich, Björn Bartling, Todd Hare, Ernst Fehr

**Affiliations:** 1 Laboratory for Social and Neural Systems Research, Department of Economics, University of Zurich, Zürich 8006, Switzerland; 2 Department of Psychology, The Ohio State University, 1827 Neil Avenue, 200E Lazenby Hall, Columbus Ohio 43210, USA; 3 Department of Economics, The Ohio State University, 1945 North High Street, 415 Arps Hall, Columbus, Ohio 43210, USA; 4 Department of Economics, University of Zurich, Zürich 8006, Switzerland

## Abstract

Do people intuitively favour certain actions over others? In some dual-process research, reaction-time (RT) data have been used to infer that certain choices are intuitive. However, the use of behavioural or biological measures to infer mental function, popularly known as ‘reverse inference’, is problematic because it does not take into account other sources of variability in the data, such as discriminability of the choice options. Here we use two example data sets obtained from value-based choice experiments to demonstrate that, after controlling for discriminability (that is, strength-of-preference), there is no evidence that one type of choice is systematically faster than the other. Moreover, using specific variations of a prominent value-based choice experiment, we are able to predictably replicate, eliminate or reverse previously reported correlations between RT and selfishness. Thus, our findings shed crucial light on the use of RT in inferring mental processes and strongly caution against using RT differences as evidence favouring dual-process accounts.

## Abstract (web summary)

In cognitive neuroscience, it is common practice to use reaction time data to infer whether decisions are intuitive or deliberate. Here the authors demonstrate that they can replicate, eliminate and reverse previously reported correlations between selfishness and reaction time.

## Graphical abstract

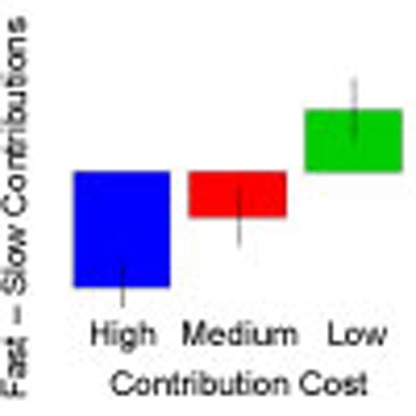

Understanding the processes behind decision-making is a fundamental goal in the social, behavioural and cognitive sciences. Across these literatures, one central question is whether behaviour is the result of a slow and deliberative process that carefully weighs the available options, or a more automatic process that is quick but prone to certain biases. A prominent view, often referred to as dual-process theory, is that both types of processes contribute to human behaviour. In the context of value-based choice, these two processes might favour different alternatives and compete to determine the decision maker’s final choice. Thus, certain decisions may come to be thought of as ‘intuitive/automatic’ (Type I), while others may be labelled as ‘deliberative’ (Type II)^1^,^2^. The distinction is important because deliberative processes should consider features of the choice problem, while intuitive processes should be insensitive to choice details. For example, giving money to a homeless person may be seen as an automatic response to help others or as a calculated action taken only when another is truly in need. Which explanation is correct has major implications for understanding human nature, and from a practical point of view for designing institutions to encourage or discourage certain behaviours. It is, therefore, crucial to identify ways to determine whether choices are intuitive or deliberative.

It has been proposed that one way to distinguish between intuitive and deliberative choices is to examine relative reaction times (RTs), the logic being that a key feature of intuitive processes is that they can be executed more quickly than deliberative processes. Decisions produced by an intuitive process should thus tend to have shorter RTs than those from a deliberative process^1^,^3^. In recent years, several researchers have used this relationship to reason backwards from RTs to infer that fast decisions are intuitive^4^,^5^,^6^,^7^,^8^,^9^,^10^,^11^,^12^,^13^,^14^,^15^,^16^. However, there are well-known pitfalls associated with making reverse inferences in other domains^17^, and a similar argument applies to RT durations. In short, there is a key distinction between the prediction that an automatic process will occur faster than more deliberative computations, and the classification of a choice as intuitive or automatic because it happens more quickly. It is well-established that various cognitive processes contribute to RT and thus any inference based on RT must account for these processes.

However, as noted above, claims relying on RT reverse inferences are all too common in the decision science literature. Again, the problem with claims based on RT correlations is that there are multiple factors that can contribute to RT. Most prominently, there is an extensive literature documenting the relationship between discriminability and RT, ranging from memory and perception^18^,^19^,^20^,^21^,^22^,^23^,^24^,^25^ to value-based/economic choice^26^,^27^,^28^,^29^,^30^,^31^,^32^,^33^,^34^,^35^,^36^,^37^,^38^,^39^. For example, in the 1990s, a now-famous debate arose over the use of RT to infer serial versus parallel visual search processes^40^. During this debate several authors demonstrated that discriminability was a key determinant of the observed RT effects, thus undermining evidence for the alternative dual-process accounts^41^,^42^. In the realm of value-based choice, decision problems involving similar options tend to take a large amount of time, while choices between dissimilar options generally take less time^26^,^29^,^30^,^33^,^35^. Therefore, it is critically important to consider the possibility that there may just be a single deliberative process governing choices, and that variations in RT are due to the perceived similarity of the choice options and not competing processes (for related points in additional domains, see^41^,^43^,^44^,^45^,^46^). In fact, if discriminability is not properly accounted for in the experimental design and/or analyses, RT asymmetries are almost guaranteed in any data set.

Here we illustrate this point in depth, using social-preference and intertemporal choice paradigms, both contexts in which others have inferred dual processes. Initially, we identify RT asymmetries between one type of choice and the other, which some might take as evidence for dual processes. However, after controlling for the strength-of-preference between choice options in these paradigms, we find no evidence that one type of response is any faster than the other. Based on these findings, we argue that modifying the choice options appropriately can produce any desired RT result (for example, fast or slow selfishness). We demonstrate this experimentally by running a replication of a public-goods experiment from a recent influential study by Rand, Greene and Nowak^5^ (henceforth, RGN), but with two additional choice problems that vary the personal cost of the pro-social act. The three different cost levels in this data set replicate, eliminate and reverse the originally observed RT asymmetries in RGN^5^. These results clearly demonstrate that RT differences or correlations should not be used as evidence for dual-process theories.

## Results

### The RT reverse-inference problem

**Figure 1:**
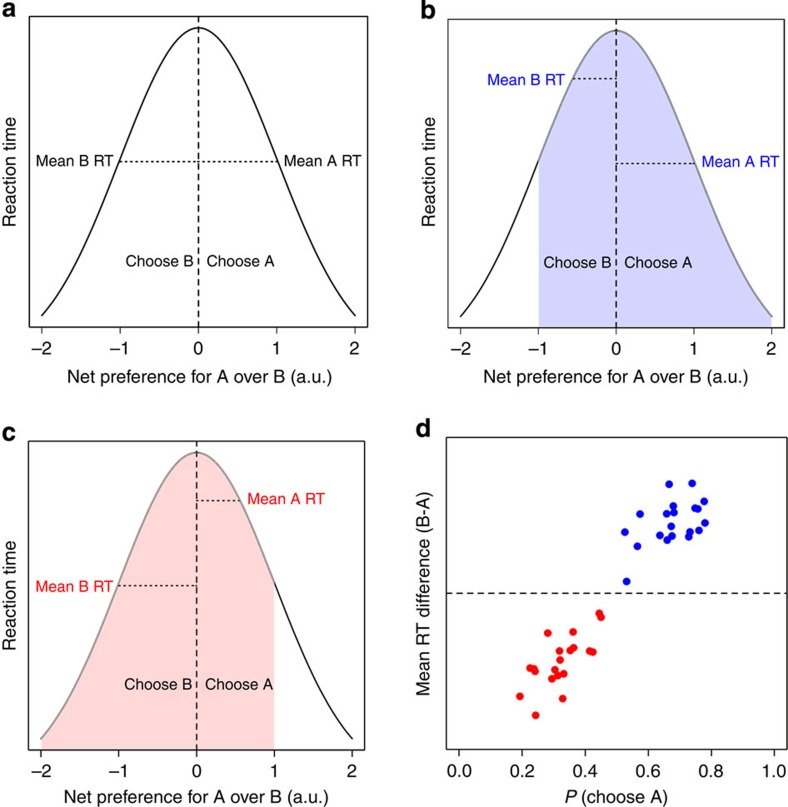
Effect of experimental design on observed RTs. Here we show a standard relationship between reaction time (RT) and the strength-of-preference between two options A and B. We assume that if the net preference for A is positive, A will be chosen over B, and *vice versa*. (**a**) If the experimenter constructs choice problems by sampling symmetrically from the left and right side of the plot (that is, A and B are equally liked on average), then A choices and B choices should on average take an equal amount of time. (**b**) If the choice set includes more options with a larger net preference for A (blue shading), A will be chosen more often, and A choices will be faster on average than B choices. (**c**) Conversely, if the choice set favours options with a larger net preference for B (red shading) B will be chosen more often, and B choices will be faster on average than A choices. (**d**) The difference in mean RT between B and A choices as a function of the overall probability of choosing A. Each dot represents one simulated subject faced with choice options drawn from either the blue shaded experiment where the net preference and probability of selecting A is greater or the red shaded experiment where the net preference and probability of selecting B is greater. The dashed line indicates an RT difference of 0. We see that choice sets near indifference (that is, *P*(choose A) =0.5) have small differences in RT between B and A choices. However, this difference in RT increases as the probability of choosing A becomes more extreme (that is, closer to 0 or 1).

We know that RT in a choice task depends critically on how different the decision maker finds the options that she is considering^47^. This is true for both perceptual and value-based decision-making. Here we will focus on value-based decision-making. Consider an abstract choice between two options A and B. If you were to plot the expected RT as a function of the difference in subjective value (preference) between A and B, you would find that the curve peaks at a value difference of 0, and falls off steadily as the strength of the preference increases in either direction (Fig. 1a).

Now imagine an experiment where subjects make multiple decisions, each time between an option from group A and an option from group B. As a concrete example, one could imagine an experiment designed to test whether people intuitively favour Ale or Bourbon. In this experiment, subjects would make a series of choices, each time between a different Ale–Bourbon pairing. The experimenter has to make a decision about which Ales and Bourbons to include in her experiment. Depending on which items she selects, she may find that the Ales she selected are generally preferred to the Bourbons. Another experimenter, running an otherwise identical experiment on the same population, may select a different set of Ales and Bourbons and find that the Bourbons he selected are generally preferred to the Ales.

The problem arises when these two experimenters compare their RT results. Experimenter 1, having a majority of trials where Ale is preferred to Bourbon, will likely have many instances where there is a strong preference for Ale and relatively fewer instances where there is a strong preference for Bourbon. This would lead to generally faster Ale choices (Fig. 1b). On the other hand, Experimenter 2 will likely have many trials where Bourbon is strongly preferred and relatively fewer trials where Ale is strongly preferred. This would lead to generally faster Bourbon choices (Fig. 1c).

Based on their results, these two experimenters, having run seemingly identical studies, would reach opposite conclusions about whether people intuitively favour Ale or Bourbon in a fast, automatic way.

One can apply the same logic to any choice task. For instance, in cooperation-game studies, we can replace A and B with Selfish and Pro-Social. The same prediction would hold. If the experiment is set-up in such a way that the pro-social options are subjectively better than the selfish options, then pro-social choices will tend to be faster. But if the experiment is slightly different, then selfish choices may be more appealing and they will tend to be faster.

There are two sources of variability in the relative attractiveness of a choice category A relative to an alternative category B. The first is due to idiosyncratic individual variability in preferences. Some subjects may generally prefer A, while others generally prefer B. If two experiments have different proportions of these subjects, they may exhibit opposite RT effects. The second source of variability is due to the choice problems selected by the experimenter as outlined above. Thus for the same set of subjects, one choice problem may strongly favour A, while another choice problem strongly favours B. Returning to our earlier example, one decision may be between a renowned craft Ale and a bottom-shelf Bourbon, while another decision may be between a generic, discount Ale and a top-shelf Bourbon.

In the following two experiments, one on social preferences and one on time preferences, we demonstrate how variability in individual preferences is systematically related to RT differences. In the third experiment, an extension of the RGN public-goods study, we demonstrate how variability in the choice problems also affects RT differences.

### Dictator game

In the Dictator Game experiment, subjects (*n*=25) in the role of the dictator made 70 binary decisions between two allocations of money, each one specifying an amount for the dictator and an amount for the receiver. For each choice, there was a selfish option and a pro-social option. Compared with the pro-social option, the selfish option gave more money to the dictator and less money to the receiver.

**Figure 2:**
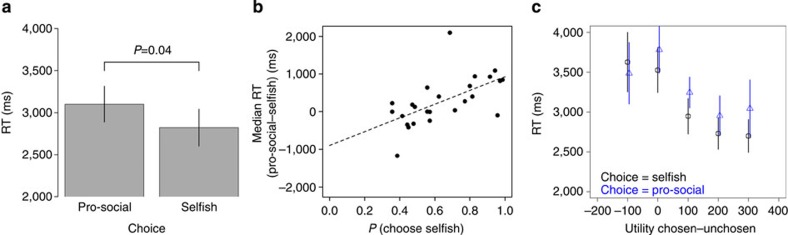
Binary Dictator Game results. (**a**) The reverse-inference result: selfish choices appear to be faster than pro-social choices, based on a paired *t*-test of the mean, across subjects, of median reaction times (RT). (**b**) The difference in median RT between pro-social and selfish choices as a function of the overall probability of choosing the selfish option. Each dot represents one subject and the dashed line is a regression line. We see that uncommon choices take more time than common choices, and that for subjects who choose each option roughly half of the time, the difference in median RT is close to 0. (**c**) RT versus utility difference, conditional on choice type. Utility difference is calculated as the difference in utility between the chosen and unchosen options, using an individually fit inequity-aversion model. After controlling for utility difference, there is no difference in RT between selfish (black) and pro-social (blue) choices. Bars represent s.e. across subjects (*n*=25).

We start by looking at RT purely as a function of choice type (pro-social versus selfish). We find that subjects were faster when choosing the selfish option (mean median: 2,822 ms) compared with the pro-social option (mean median: 3,100 ms, *t*(24)=2.16, *P*=0.04) (Fig. 2a). On this basis we might conclude that selfish decisions are fast and intuitive, while pro-social choices are more deliberative.

It is important to note that each trial has a different tradeoff between what the dictator has to personally give up and how much he benefits the receiver by choosing the pro-social option. In some trials the dictator has to give up very little in exchange for a big gain for the receiver, but in other trials the dictator has to give up a lot in exchange for a small gain for the receiver, while a third type of trial falls somewhere in between. We refer to the first type of trials as ‘high-benefit’ trials, and to the second type of trials as ‘low-benefit’ trials.

So far we have only discussed objective tradeoffs, that is, dollar cost to the dictator versus dollar benefit to the receiver. For any given trial, these tradeoffs are identical across subjects. But of course, subjects may differentially value money for themselves compared with money for others. ‘Selfish’ subjects place relatively low value on money for others, while ‘pro-social’ subjects place relatively high value on money for others. For any given trial, a selfish subject will thus be more likely to choose the selfish option than his pro-social counterpart.

Based on the arguments laid out above, we would expect that in a given experiment a selfish subject (that is, one who primarily chooses the selfish option in this experiment) would tend to make faster selfish decisions and slower pro-social decisions. The more selfish the subject is, the harder it will be for him to be pro-social. Thus, more extreme selfishness will result in a larger difference between pro-social and selfish RTs. Similarly, a pro-social subject (again defined by his decisions in the experimental choice set) would tend to make slower selfish decisions and faster pro-social decisions. That is, the more extreme the pro-sociality, the bigger the gap between selfish and pro-social RTs.

Turning to the data, we indeed find a strong correlation between a subject’s probability of choosing the selfish option (that is, his degree of selfishness) and the difference between his median RT for pro-social and selfish choices (*r*=0.6, *t*(23)=3.56, *P*=0.002) (Fig. 2b). Furthermore, note that at indifference (*P*(choose selfish)=0.5) the difference between median RTs is ∼0. In other words, if our experiment is perfectly designed to make our subject choose each option half of the time (and with equal vigour), then we should find no difference in RTs. However, such experiments are rare due to the fact that they must be tailored to each individual.

In our experiment, the bias was clearly towards low-benefit trials because subjects chose the selfish option 64% of the time, yielding selfish choices that were faster than the pro-social choices. Thus, the RT difference observed in these experiments is likely a result of the fact that subjects chose the selfish option more than half of the time.

To control for choice difficulty when examining RT, we applied a well-established model of social preferences developed by Fehr–Schmidt and later Charness–Rabin, to estimate each subject’s preference for pro-social acts^48^,^49^. This utility function allows us to convert each two-dimensional choice option (dictator payoff and receiver payoff) into one subjective value. Specifically, we use the following utility function:




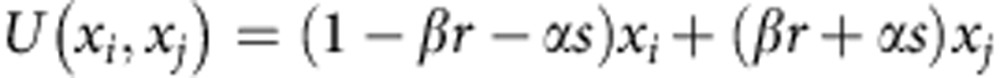




where *x*_*i*_ is the payoff to the dictator, *x*_*j*_ is the payoff to the receiver, *r* and *s* are dummy variables for whether the dictator’s payoff is higher or lower than the receiver’s payoff, respectively, and *β* and *α* are individually fit preference parameters for each of those contingencies, respectively. We can then use the difference in utility between the chosen and unchosen options as an index of the strength-of-preference.

Next, we analysed RTs as a function of both choice type (selfish versus pro-social) and the difference in utility between the two choice options. Figure 2c displays two features of the data. First, mean RT decreases as the utility difference increases, that is, as one option becomes increasingly better than the other. Second, at each level of utility difference, there is no difference in RT between selfish and pro-social choices. What drives the overall RT difference in the data set is that there are simply more trials where there was a high utility-difference advantage for the selfish option.

To carefully test these observations, we conducted a mixed-effects regression with log(RT) as the dependent variable explained by independent variables for utility difference and a dummy for pro-social choices. These regressions revealed significant effects of utility difference (*t*(1,740)=7.37, *P*<0.001) but no effect of the dummy for pro-social choice (*t*(1740)=0.87 *P*=0.38) on RT. In other words, the potential conclusion that selfish decisions are fast and intuitive is merely an artifact of the parameters of the experiment. Once we correct for the strength-of-preference in each trial, using utility differences, there is no evidence that selfish or pro-social choices take different amounts of time.

### Intertemporal choice

So far we have demonstrated that after taking choice difficulty into account, our data show no difference in RT for self-centered versus other-regarding choices. However, claims of competing dual processes in decision-making are not limited to the domain of social preferences. For example, some have argued that different processes govern choices between immediate and delayed rewards^50^,^51^,^52^ (but see the studies by Kable and Glimcher^53^,^54^). If this were the case, one might expect to see RT differences between such choices.

To investigate this possibility, we analysed a temporal-discounting data set where 41 subjects made 216 binary choices between $25 now and some larger amount *x, t* days in the future^55^. In brain-imaging studies like this one, it is often important to have a balanced design. In this study, subjects chose the immediate $25 option on 53% of trials, which was not significantly different from 50% (*t*(40)=0.88, *P*=0.39). On that basis, we should expect no significant difference in RT for immediate versus delayed choices. Indeed, we find no difference in RT for immediate choices (1,169 ms) compared with delayed choices (1,229 ms, *t*(40)=1.46, *P*=0.15), though the difference goes in the expected direction, with the slightly less-favoured delayed choices being slightly slower.

For the sake of argument, let us suppose that the authors had not been so careful with their design, or had, for instance, wanted more trials where the immediate option would be chosen. Thus, let us examine a subset of the full experiment, focusing on trials where the immediate option is quite attractive compared with the delayed option and so we would have predicted more choices of the immediate option, based on median preferences reported in previous experiments^53^ (see Methods).

**Figure 3:**
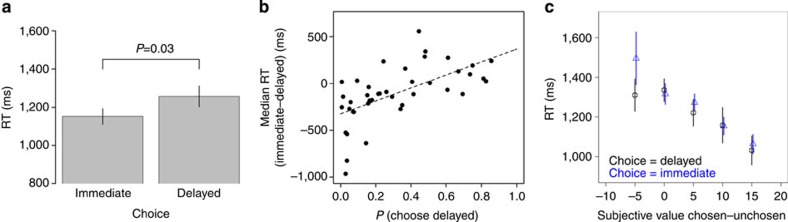
Intertemporal choice results. (**a**) The reverse-inference result: choices of the immediate option appear to be faster than choices of the delayed option, based on a paired *t*-test of the mean, across subjects, of median RT. (**b**) The difference in median RT between immediate and delayed choices as a function of the overall probability of choosing the delayed option. Each dot represents one subject and the dashed line is a regression line. We again see that uncommon choices take more time than common choices, and that for subjects who choose each option roughly half of the time the difference in median RT is close to 0. (**c**) RT versus subjective-value difference, conditional on choice type. Subjective-value difference is calculated as the difference in subjective value between the chosen and unchosen options, using an individually fit hyperbolic-discounting model. After controlling for subjective-value difference, there is no difference in RT between delayed (black) and immediate (blue) choices. Bars represent s.e. across subjects (*n*=41).

In this reduced data set, we do find an overall difference in RT for immediate choices (1,152 ms) compared with delayed choices (1,257 ms, *t*(40)=2.22, *P*=0.03) (Fig. 3a). On this basis we might conclude, consistent with a large fraction of the literature, that choices of the immediate payoff are fast and intuitive, while choosing to wait for a bigger payoff is slow and deliberative.

However, just as in the social decisions, there is a strong correlation between a subject’s probability of choosing the delayed option and the difference in the median RT between choices for the immediate and delayed options (*r*=0.61, *t*(39)=4.79, *P*=10^−5^) (Fig. 3b). In other words, impulsive subjects take more time when they choose the delayed option, while patient subjects take more time when they choose the immediate option. The overall RT effect in the reduced data set is simply due to the fact that there are many more trials where choosing the immediate option is easy for everyone (compared with easy-delayed trials), and so choices of the immediate option are more common (68%) and faster.

As in the Dictator Game analysis, we next applied a model of temporal discounting to control for strength-of-preference when analysing RTs. To do so, we converted the delayed reward into its present discounted value, using the standard hyperbolic-discounting function:




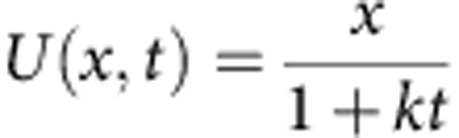




where *k* is a subject-level parameter (fit to each subject using a maximum likelihood procedure, see methods) that determines how much he discounts future rewards. We then used the difference between the discounted subjective value of the delayed option and the value of the immediate option ($25) as an index of strength-of-preference.

We next analysed RTs as a function of both choice type (delayed versus immediate) and the difference in value between the two choice options. Figure 3c displays the same two features of the data that we observed in the Dictator Game experiment. First, mean RT decreases as the value difference increases, that is, as one option becomes increasingly better than the other. Second, at each level of value difference, there is no difference in RT between delayed and immediate choices.

These observations are again supported by a mixed-effects regression analogous to the one computed for the Dictator Game, which shows a significant effect of value difference (*t*(5,576)=9.27, *P*<0.0001) on log(RT), but no effect of choice type (immediate versus delayed) (*t*(5,576)=0.24, *P*=0.81). Note that the results are analogous when analysing the full data set.

### Arbitrary generation of RT asymmetries

Recently, a high-impact study by RGN claimed that human altruistic cooperation is governed by a dual-process mechanism that pits a fast and intuitive system favouring cooperation against a slow, calculating system favouring selfishness^5^. This claim was based on the results of several social cooperation experiments (prisoners’ dilemma and public-goods games (PGG)) conducted by the authors. In the PGG experiments that are the primary focus of their main text, subjects were anonymously assigned to groups of 4 and each was given an endowment of 40 money units (MU). Subjects then decided how many MUs to keep, and how many to contribute to the public good. Any contributed MUs were doubled by the experimenter and split evenly among the four group members. Thus, each subject in the group received 0.5 MU for each 1 MU contributed by a group member.

In their original study, RGN found that subjects who contributed less to the public good responded more slowly, while faster responders contributed more MUs. The authors interpreted these findings as suggesting that intuitive responses are more cooperative (that is, pro-social) and used these findings to motivate additional experiments using priming and time interventions as more direct tests of the pro-social tendencies suggested by the RT correlations. Moreover, in subsequent work they and others have developed a more elaborate theory specifying when and for whom intuition should favour cooperation as opposed to selfishness^8^. In this regard, we wish to point out that the relationship between RT and strength-of-preference does not preclude possible biases or tendencies towards pro-social or myopic behaviour that might be exacerbated by time pressure or priming, but it does prohibit drawing conclusions about fast, intuitive decision mechanisms based on RT correlations. We utilize the RGN style PGG as an example here, not to argue against a tendency for pro-social behaviour, but because the set-up of the game makes it very simple to change the attractiveness of pro-social behaviour and directly demonstrate the relationship between RT and strength-of-preference.

The PGG is very similar to the previously discussed Dictator Game. Each subject is faced with the following tradeoff: keep each MU or keep 0.5 of each MU and give 0.5 to three other subjects. Now imagine two alternative versions of their experiment, A1 which yields a benefit of 0.9 MU per subject for each contributed unit, and A2 which yields just 0.3 MU per subject for each contributed unit. In A1, the purely selfish option keeps 40 for oneself and gives 0 to each of the three others, while the purely pro-social option produces 36 for oneself and 36 for each of the others. In A2, the purely selfish option is the same as before, but the purely pro-social option only produces 12 for one oneself and 12 for each of the others. Thus, in the first setting, contributing is minimally costly and maximally beneficial to others, while in the second setting contributing is very costly and minimally beneficial to others.

Using the terminology that we introduced earlier, A1 is a high-benefit choice problem, whereas A2 is a low-benefit choice problem. As in the Dictator Game, the prediction from the strength-of-preference account is that in A1 the selfish subjects will be slow and indecisive while the pro-social subjects will be quick and cooperative, whereas in A2 the pro-social subjects will be slow and indecisive while the selfish subjects will be quick and uncooperative. On average, this will result in relatively fast pro-social behaviour in A1, but relatively slow pro-social behaviour in A2.

To test these predictions, we conducted a replication of the RGN public-goods experiment, using their original benefit level (0.5 MU per subject for each contributed unit), as well as the two alternative levels described above (0.9 MUs per subject and 0.3 MUs per subject). Each subject (*n*=175) made three decisions total, one for each benefit level.

The dual-process and strength-of-preference accounts yield different predictions in this experiment. On the one hand, the dual-process account predicts that in all three versions of the experiment we should see a negative correlation between RT and contribution amount. On the other hand, we predicted that when the overall contribution amount is <50% (contributing is the less attractive choice) there should be a positive correlation between RT and contributions, but when the overall contribution amount is >50% (contributing is more attractive) there should be a negative correlation between RT and contributions.

As expected from the literature^56^, varying the group benefit from the public good influenced subjects’ average contribution rates, with subjects’ contributions increasing from 25% to 47% (*t*(174)=5.87, *P*=10^−8^) to 63% (*t*(174)=3.9, *P*=0.0001) with benefits of 0.3, 0.5 and 0.9, respectively.

**Figure 4:**
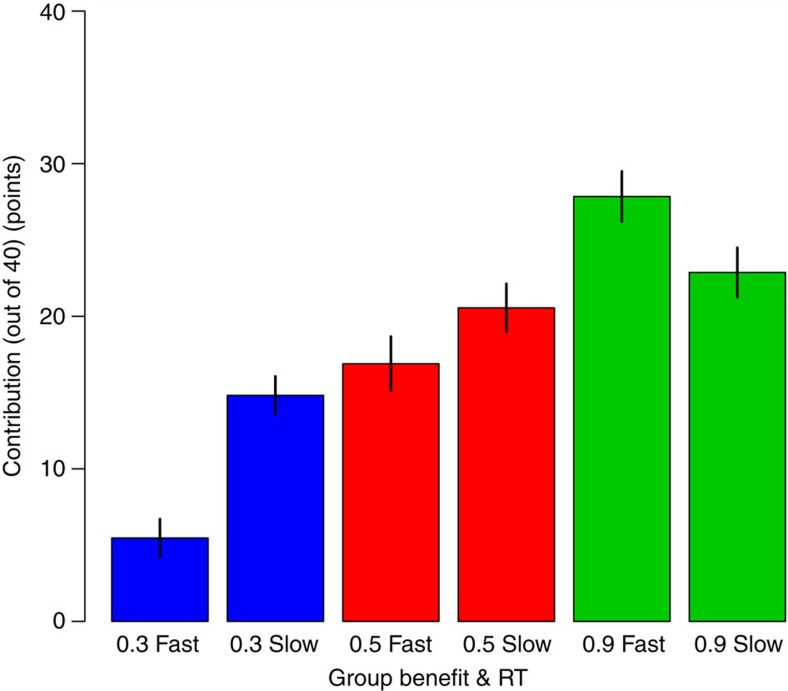
Public-goods results. Analogous to RGN’s (Fig. 1a), we plot the contribution amounts for the fast and slow half of the subjects, here based on a median split within each experiment (*n*=175 per experiment). As predicted by the strength-of-preference account, with a small group benefit we see that the slow subjects contribute more (two-sided *t*-test, *P*=10^−5^), whereas with a large group benefit we see the opposite; fast subjects contribute more (two-sided *t*-test, *P*=0.03). At the intermediate group-benefit level, we see a small trend towards significance (two-sided *t*-test, *P*=0.12), which is to be expected based on the overall contribution rate just slightly <50%.

Consistent with the strength-of-preference predictions, the quicker half of the subjects in the 0.3 MU per subject treatment contributed less (14%) than the slower half (38%, *t*(158)=4.49, *P*=10^−5^), while in the 0.9 MU per subject treatment the quicker half of the subjects contributed more (70%) than the slower half (58%, *t*(172)=2.16, *P*=0.03) (Fig. 4). For the original 0.5 MU per subject treatment, we find a trend towards significance in the opposite direction to RGN, namely that the quicker half of the subjects contribute less (43%) than the slower half (53%, *t*(169)=1.54, *P*=0.12).

Analysing the data another way, we find a positive Spearman’s correlation between RT and contributions in the treatment where the benefit is 0.3 (*r*=0.34, *P*=10^−6^), a marginally positive correlation in the treatment with the benefit of 0.5 (*r*=0.1, *P*=0.18), and a negative correlation in the treatment with the benefit of 0.9 (*r*=−0.15, *P*=0.045).

From the strength-of-preference perspective, the discrepancy between the RGN results and our results in the 0.5 MU per subject treatment is not surprising because in the RGN data set subjects contributed slightly >50%, predicting quick contributions, while in our data set the subjects contributed slightly, though not significantly <50% (*t*(174)=1.17, *P*=0.24), predicting slower contributions.

To summarize, contrary to the dual-process prediction of a negative correlation between RT and cooperation in all cases, we observe a positive correlation between RT and cooperation in two out of three treatments. Importantly, these seemingly mixed results are perfectly consistent with the strength-of-preference account.

## Discussion

We have demonstrated a robust relationship between RT and choice probabilities, and the importance of taking strength-of-preference into account when making inferences based on RT data. By controlling for subjective-value differences, we showed that there were no RT differences between selfish and pro-social choices in a social-preference data set, and similarly that there were no RT differences between patient and impatient choices in a temporal-discounting data set. These findings highlight the pitfalls of using RT correlations as support for dual-process theories.

In the specific case of social preferences, our results help to resolve a debate in the literature where some authors find positive correlations between RT and pro-sociality, while others find the opposite. We argue that which response is faster will depend critically on the parameters of the decision problem. If it is costly to contribute, as in our low-benefit treatment, then being selfish is the faster response, whereas if it is cheap to contribute, as in our high-benefit treatment, then being pro-social is the faster response.

We want to emphasize again that these results are not incompatible with the existence of a general bias towards pro-social behaviour. Our arguments and results do not directly address the use of data from time-pressure and priming experiments or the debates surrounding those results^5^,^8^,^57^,^58^,^59^,^60^,^61^,^62^,^63^,^64^,^65^,^66^. In the results presented here, we do find a bias in the opposite direction, with <50% contributions in two out of three of our conditions. However, it is also important to note that the presence of a choice-bias alone does not imply a dual-process mechanism. Various single-process choice models including evidence accumulation models and other forms of Bayesian updating mechanisms explicitly allow for response biases in the choice process while simultaneously accounting for RTs and the influence of varying decision parameters (for example, different group-benefit levels) on choices^25^.

In summary, using decision timing to make inferences about whether certain behaviours are governed by the fast and intuitive component(s) of a dual-process model is problematic and often misleading if the values of underlying choice options are not properly accounted for. We have shown here that asymmetries in RT between choice types in decision contexts often suggested to involve competition between fast, intuitive and slow, deliberative processes (for example, moral, social, and intertemporal) can be explained by differences in the strength-of-preference or discriminability between choice options. Our results highlight the need for more careful treatment of RT data when adjudicating between competing models of decision-making.

## Methods

### Dictator game experiment

The Dictator Game behavioural data is from a functional magnetic resonance imaging (fMRI) experiment conducted at the University of Zurich’s Social and Neural Systems laboratory. Subjects (*n*=30) were asked to make a choice between two possible allocations of money, option X and option Y, where there was a tradeoff between their own payoff and the receiver’s payoff. Five subjects were excluded from the analysis because they always chose the selfish option precluding a comparison of RTs between the two choice types. The data from the remaining 25 subjects is presented here. Subjects also completed 50 additional trials that did not include a tradeoff between self and other payoff and so we do not analyse those trials here. All subjects gave written informed consent. The study was approved by the ethics committee of the Canton of Zurich.

The choice problems were presented in a random sequence, and the subjects were asked to make their decisions within 10 s. Subjects first observed a screen that announced the upcoming Dictator Game (‘Choose X or Y’), followed by a decision screen that included the options X and Y. After the subjects made their choice using a two-button MRI-compatible button box, a fixation cross appeared in the centre of the screen during the inter-trial interval. If the subject did not make a decision within 10 s, then a choice was automatically made for the fair option and the experiment moved forward to the inter-trial interval. Three trials were excluded for failing to fall within this time limit. Inter-trial intervals varied between 3 and 7 s.

Prior to scanning, subjects read written instructions describing the task and the payoff rules. Comprehension of the payoff rules and the treatment conditions was tested by means of a control questionnaire. All subjects answered the control questions correctly and thus knew that they played with anonymous human interaction partners and that their decisions were treated in an anonymous way. The overall payment to the participants consisted of a fixed show-up fee (25 Swiss Francs (CHF)) plus the payment from six randomly selected choice problems. On average, participants earned 65 CHF (ranging from 55 to 79 CHF).

### Temporal-discounting experiment

We analysed the choices and RTs from 41 individuals, a subset of whom (*n*=27) were included in a previously reported fMRI analysis^55^. The additional 14 subjects included here represent behavioural pilots and subjects for whom the fMRI data could not be used. All subjects gave written informed consent. The study was approved by Caltech’s Internal Review Board. Subjects made 216 choices between getting $25 at the end of the experiment or getting an equal or larger amount at a later date. The delayed offers ranged from $25 to $54, with a delay from 7 to 200 days. Each trial began with an offer presented onscreen. Participants were required to press a button within 3 s to indicate whether or not they accepted the delayed reward being offered. Only the varying delayed option was presented onscreen. A button press response resulted in the termination of the offer screen, and the appearance of a feedback screen for 250 ms displaying ‘Yes’, if the delayed offer was accepted, or ‘No’, if it was rejected. The phrase ‘No decision received’ was displayed if the subject failed to respond within 3 s (the mean no response rate across subjects was 2.6% of trials). Trials were separated by a fixation cross of random duration (uniform: 2–6 s). At the end of the experiment, a single trial was randomly chosen and implemented: subjects received the chosen option in addition to $50 (available immediately) for participating in the study. All payments were made using prepaid debit cards given to the subjects at the end of the experiment. This allowed us to make the delayed payments available on the appropriate date, without requiring subjects to return to the lab.

### Hyperbolic-discounting model fits

We estimated an individual discount factor (denoted by *k*) for each subject using maximum likelihood estimation. In particular, we assumed that subjects assigned value to the delayed options using a hyperbolic-discounting function, in which the utility of *x* dollars with a delay of *t* days is given by the equation:




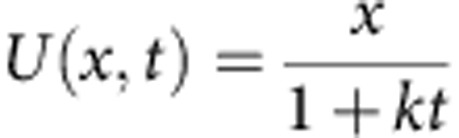




We also assumed that the probability of accepting the delayed option is given by the soft-max function




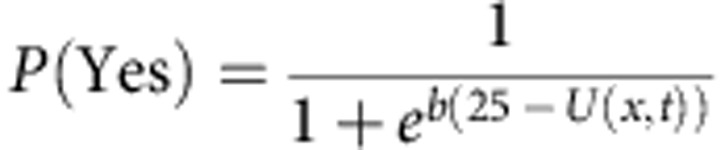




where *b* is a non-negative parameter that modulates the slope of the psychometric choice function. In this formula the value of the constant reference option is $25. Note that the fits were performed using the full data set for each subject.

### Reduced data set

To produce the reduced data set, we utilized the same hyperbolic-discounting function described above. To independently select a subset of the trials with which to demonstrate our point concerning RT inferences, we took the median *k* value (*k*=0.01 per day) from a similar study on temporal discounting^53^. We then removed all trials in which the utility of the delayed option was <$25. This reduced the data set from 216 trials to 140 trials per subject.

### Public-goods experiment

We recruited 204 non-economics students who had no experience with PGG from the regular subject pool of the decision laboratory of the Department of Economics at the University of Zurich, where the sessions took place in May and June 2013. The sample size was chosen to roughly match the sample size in RGN. All subjects gave written informed consent. The study was approved by the ethics committee of the Canton of Zurich. The experiment was computerized with the software z-Tree^67^. Subjects first received general details about the game, shown on the first computer screen. On the subsequent decision screens, subjects were told the respective group benefit from the public good, and how much they would earn if the others in their group contributed everything and if they either contributed everything or nothing (as in RGN). They could then type in an integer contribution from 0 to 40 points (2 points=1 CHF) and then press an ‘OK’ button on the screen. RT was calculated as the time from the onset of the decision screen to the time that subjects clicked on the ‘OK’ button.

Each subject made three such decisions, one for each group-benefit level (0.3, 0.5 and 0.9 MU per subject), in a counterbalanced order. After all three decisions, subjects were given two incentivized comprehension questions asking for the efficient contribution and the contribution that maximizes own earnings (as in RGN). 175 subjects successfully answered both questions and the rest were excluded from subsequent analysis.

At the end of the study, subjects were randomly assigned to groups of four and paid according to their choices from one of the three randomly selected games. This was the only time that subjects received any feedback about others’ choices. The overall payment to the participants consisted of a fixed show-up fee (10 CHF) plus the payment from the randomly selected PGG. On average, participants earned 27.55 CHF (ranging from 12.40 to 44.60 CHF). Sessions lasted for a little less than 1 h, including the payment of the subjects.

### Simulations

We simulated 20 ‘subjects’ for each experiment (Fig. 1b,c). For each subject, we randomly selected a logit temperature parameter from a uniform distribution between 0.1 and 10, and we also randomly selected 20 choice trials from a uniform distribution over the net preference values given in Fig. 1b,c, depending on the experiment. We then determined the average probability of choosing A for that simulated subject across its 20 choice trials. For each subject, we determined the average RT for A and B choices by looking at each trial and multiplying the probability of making either choice by the appropriate RT depicted in Fig. 1a–c. We then computed the normalized sum of these weighted RTs across the 20 choice trials, once for the A choices and once for the B choices.

## Additional information

**How to cite this article**: Krajbich, I. *et al.* Rethinking fast and slow based on a critique of reaction-time reverse inference. *Nat. Commun.* 6:7455 doi: 10.1038/ncomms8455 (2015).
